# Ectocervical Hypergranulation Secondary to Transcervical Coil Thread Perforation—An Unusual Cause for Persistent Postcoital Bleeding

**DOI:** 10.1002/ccr3.72327

**Published:** 2026-03-19

**Authors:** Negin Sadeghi, Karin Hellner

**Affiliations:** ^1^ Department of Gynaecology Oxford University Hospitals NHS Foundation Trust Oxford UK; ^2^ Nuffield Department of Women's & Reproductive Health, John‐Radcliffe Hospital, Women's Center University of Oxford Oxford UK

**Keywords:** cervix, coil, colposcopy, postcoital bleeding, vaginal bleeding

## Abstract

Cervical perforation by coil threads is an uncommon complication. Predisposing factors may include prior cervical interventions, hypoestrogenic atrophy, thread material, and duration of use. Awareness is essential when evaluating abnormal bleeding or atypical thread presentation despite radiologically correct placement.

## 1 Case Presentation

A 36‐year‐old multiparous woman (P2) with a history of LLETZ for cervical intraepithelial neoplasia (CIN) was referred to colposcopy for irregular vaginal bleeding. She has a diagnosis of premature ovarian insufficiency and had recently started transdermal estradiol therapy. She had a Mirena levonorgestrel‐releasing intrauterine system (LNG‐IUS) inserted 3 years prior and initially presented with persistent postcoital bleeding to her gynecologist. She has no other significant medical history. Her last cervical cytology was recent and reported as normal with no presence of high‐risk human papillomavirus infection. A recent ultrasound confirmed correct position of the IUS.

**FIGURE 1:**
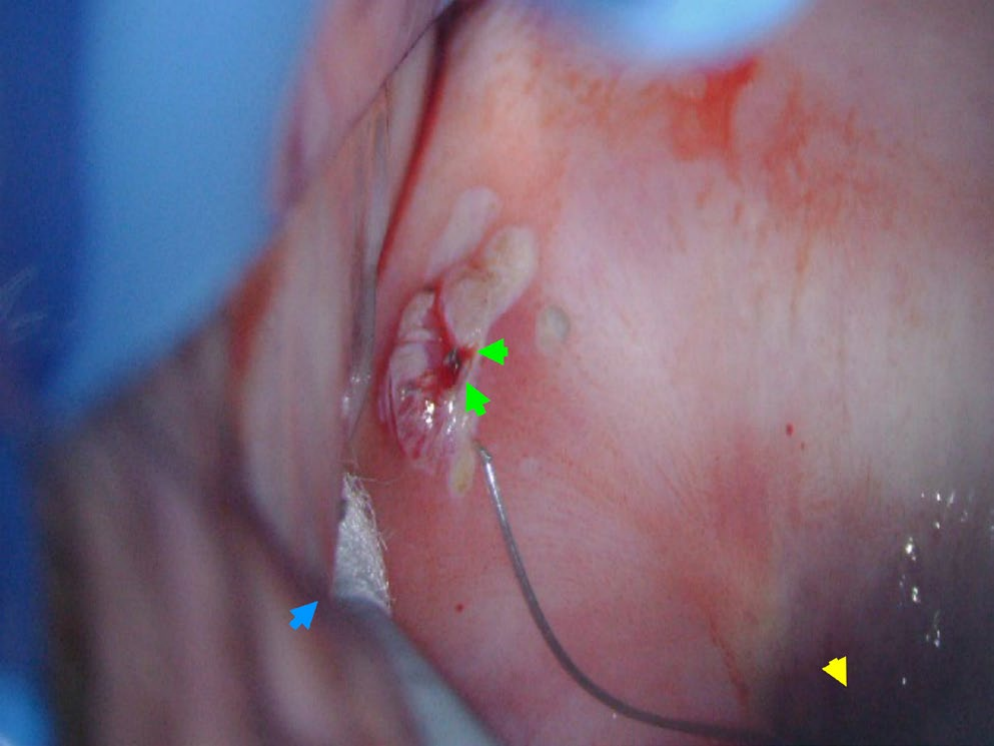
Colposcopic view showing ectocervical perforation. The green arrows indicate the IUS thread end which perforated through the ectocervical periphery. The second—correctly sited—thread is seen posterior to this (yellow arrow). The blue arrow shows a cotton swab that was used to stabilize the cervix during colposcopy.

Colposcopy revealed a polypoid, fleshy ectocervical lesion, which was bleeding heavily upon touch. Application of acetic acid showed faint acetowhiteness of the raised lesion, but no features of CIN or a malignancy (Figure 1). Closer inspection and gentle manipulation of the cervix with a cotton bud demonstrated a coil thread perforating through the ectocervical tissue, ~2 cm from the os, with surrounding granulation tissue (Video 1). Cauterization with Silvernitrate in clinic was unsuccessful and she was referred for IUS removal and cervical cautery under general anesthesia.

**VIDEO 1:** Thread position relative to the cervical os. Shown is a short video of the colposcopy, which highlights the perforation site with the thread end visibly moving back and forth during manipulation of the cervix with a cotton swab. Video content can be viewed at https://onlinelibrary.wiley.com/doi/10.1002/ccr3.72327.

## 2 Discussion

Cervical perforation by intrauterine device (IUD) strings is an exceedingly rare complication, distinct from uterine body or fundal perforation. A recent systematic review identified only eight published case reports that fulfilled strict inclusion criteria of string penetration through cervical tissue without associated uterine perforation [1]. The mean patient age was 35.4 ± 7.8 years (range 26–47 years), with both copper IUDs (*n* = 5; 62.5%) and LNG‐IUS (*n* = 3; 37.5%) implicated [1]. Gravidity varied, but 12.5% were nulligravid and 37.5% multigravida. 87.5% of patients were asymptomatic, with the perforation often detected incidentally during routine gynecological examination [1].

The mechanism of thread penetration remains unclear, but proposed theories include:

- Gradual erosion of cervical epithelium by mechanical friction of taut strings, particularly in cases with low cervical position of the IUD.
- Iatrogenic trauma at insertion causing micro perforations, with subsequent fibrous incorporation of the threads.
- Postpartum cervical changes predisposing to tissue vulnerability [1].

In most reported cases, minor surgical manipulation (e.g., gentle traction, forceps repositioning, or hysteroscopic assistance) allowed successful repositioning of the strings into the endocervical canal without device removal [1].

Given the scarcity of cases and absence of large case series, this complication is likely underreported. Nevertheless, clinicians should consider it in the differential when coil threads are not visualized at the external os but are seen protruding from an abnormal site on the cervix, even in asymptomatic patients.

## 3 Teaching Points

- Cervical perforation by IUD threads is an uncommon complication.
- Predisposing factors may include prior cervical interventions, hypoestrogenic atrophy from POI, thread material, and duration of use (e.g., LNG‐IUS now approved for up to 8 years).
- Awareness is essential when evaluating abnormal bleeding or atypical thread presentation despite radiologically correct placement.

## Author Contributions

**Negin Sadeghi:** data curation, investigation, methodology, visualization, writing – original draft, writing – review and editing. **Karin Hellner:** conceptualization, project administration, supervision, visualization, writing – review and editing.

## Funding

The authors have nothing to report.

## Ethics Statement

The authors have nothing to report.

## Consent

Written informed consent to publish this report was obtained from the patient. This is available upon request from the corresponding author.

## Data Availability

Data is available from the corresponding author.
